# Supplementary material for: Strategies for Pain Management in Hepatocellular Carcinoma Patients Undergoing Transarterial Chemoembolisation: A Scoping Review of Current Evidence
Source: Healthcare (Basel). 2025 Apr 25;13(9):994. doi: 10.3390/healthcare13090994 (PMC12071419; doi:10.3390/healthcare13090994)

Search Strategy

Pubmed:

(((((pain) OR (pain management)) OR (pain control)) OR (analgesia)) AND ((TACE) OR (transarterial chemoembolization))) AND (((HCC) OR (hepatocellular carcinoma)) OR (liver cancer))

Search	Actions	Details	Query	Results	Time
#9	•••	>	Search: **(((((pain) OR (pain management)) OR (pain control)) OR (analgesia)) AND ((TACE) OR (transarterial chemoembolization))) AND (((HCC) OR (hepatocellular carcinoma)) OR (liver cancer))**	451	06:40:18
#8	•••	>	Search: **((HCC) OR (hepatocellular carcinoma)) OR (liver cancer)**	375,270	06:39:56
#7	•••	>	Search: **(TACE) OR (transarterial chemoembolization)**	10,152	06:39:01
#6	•••	>	Search: **(((pain) OR (pain management)) OR (pain control)) OR (analgesia)**	1,111,442	06:38:28

Web of Science:

(((TS=(pain)) OR TS=(pain management)) OR TS=(pain control)) OR TS=(analgesia) AND (TS=(TACE)) OR TS=(transarterial chemoembolization) AND ((TS=(HCC)) OR TS=(hepatocellular carcinoma)) OR TS=(liver cancer)

☐	5	**#4** and **Preprint Citation Index** (Exclude – Database)	646
☐	4	**#3 AND #2 AND #1** and **Preprint Citation Index** (Exclude – Database)	646
☐	3	**((TS=(HCC)) OR TS=(hepatocellular carcinoma)) OR TS=(liver cancer)** and **Preprint Citation Index** (Exclude – Database)	516,661
☐	2	**(TS=(TACE)) OR TS=(transarterial chemoembolization)** and **Preprint Citation Index** (Exclude – Database)	17,502
☐	1	**(((TS=(pain)) OR TS=(pain management)) OR TS=(pain control)) OR TS=(analgesia)** and **Preprint Citation Index** (Exclude – Database)	1,448,871

Cochrane Library:

Print search history

#1	(pain):ti,ab,kw OR (pain management):ti,ab,kw OR (pain control):ti,ab,kw OR (analgesia):ti,ab,kw<br>(Word variations have been searched)	Limits	267382
#2	(TACE):ti,ab,kw OR (transarterial chemoembolization):ti,ab,kw<br>(Word variations have been searched)	Limits	1628
#3	(HCC):ti,ab,kw OR (hepatocellular carcinoma):ti,ab,kw OR (liver cancer):ti,ab,kw<br>(Word variations have been searched)	Limits	18027
#4	#1 AND #2 AND #3	Limits	162

CINAHL:

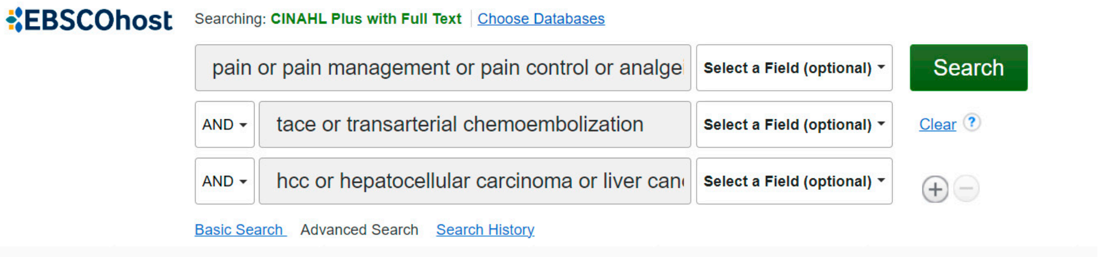

Scopus:

TITLE-ABS-KEY (pain OR pain AND management OR pain AND control OR analgesia) AND TITLE-ABS-KEY (tace OR transarterial AND chemoembolization) AND TITLE-ABS-KEY (hcc OR hepatocellular AND carcinoma OR liver AND cancer)

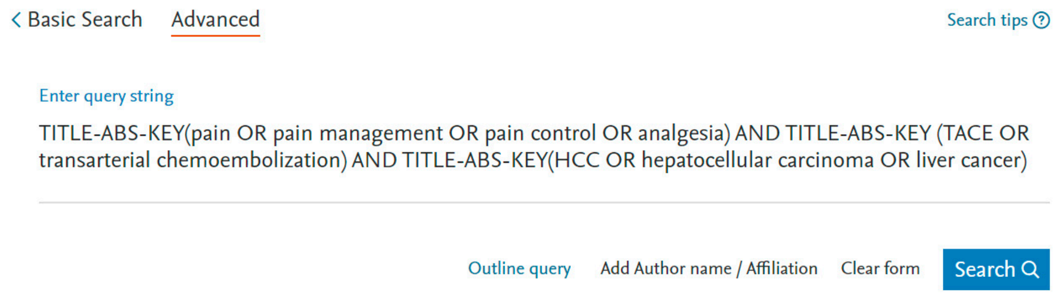

Supplement: Supplementary file 1 [file healthcare-13-00994-s001.zip › healthcare-3552969 - Supplementary material S2-Search strategy.pdf]
